# Reducing COVID-19 Vaccine Decisional Conflict in Parents of 5–11-Year-Old Children in Australia: A Single Arm Pre-Post Study

**DOI:** 10.3390/vaccines11081296

**Published:** 2023-07-28

**Authors:** Zephaniah Hilton, Monsurul Hoq, Margie Danchin, Jessica Kaufman

**Affiliations:** 1 Vaccine Uptake Group, Murdoch Children’s Research Institute, Parkville 3052, Australiamargie.danchin@rch.org.au (M.D.); jess.kaufman@mcri.edu.au (J.K.); 2 The National Child Health Poll, The Royal Children’s Hospital, Parkville 3052, Australia; 3 Clinical Epidemiology and Biostatistics Unit, Murdoch Children’s Research Institute, Melbourne 3052, Australia; 4 Department of General Medicine, The Royal Children’s Hospital, Parkville 3052, Australia; 5 Department of Paediatrics, The University of Melbourne, Parkville 3052, Australia

**Keywords:** COVID-19, vaccine hesitancy, vaccination, shared decision making, decisional conflict

## Abstract

COVID-19 vaccine uptake among 5–11-year-olds is significantly lower than that of the 12+ age group. Some parents may have decided against vaccinating their children for COVID-19; others may be undecided and may be seeking more information to support their decision. We aimed to assess the effect of a decision support tool on parents’ level of decisional conflict, vaccine hesitancy, and intention to vaccinate. We conducted a single-arm, cross-sectional online pre-post intervention survey of parents from Victoria, Australia, who had not yet vaccinated their 5–11-year-old child for COVID-19. We measured change in decisional conflict, intention, and hesitancy towards COVID-19 vaccines for children before and after viewing a decision support tool. We used logistic regression to identify characteristics associated with reduced decisional conflict. Between May and September 2022, 108 parents took part in the study. The tool reduced decision conflict in 25% (27/107) of parents, with reduced decisional conflict more likely among parents initially undecided about vaccinating, compared to parents who did not intend to vaccinate their child (OR, 12.58 95% CI 3.21 to 9.30). For most parents, hesitancy (83%, 90/108) and intention (89%, 96/108) remained the same. The decision support tool was modestly effective at reducing decisional conflict, particularly among undecided parents.

## 1. Introduction

COVID-19 is less severe in children than adults [1,2]. However, some children with COVID-19 still require hospitalization and experience serious consequences, especially those with certain underlying medical conditions that place them at higher risk [3]. The risk-benefit equation for vaccination is less conclusive in children than adults; however, vaccination still provides direct and indirect benefits, including protection against the long-term consequences of COVID-19 infection [2,4,5]. Australia initiated the vaccine rollout for 5–11-year-old children on January 10, 2022. While uptake was initially strong, it plateaued after several weeks and remained at 50% one year later. Other countries, including Canada, the US, and the UK, have also experienced low vaccine coverage in this age group relative to older population groups [5].

Slow vaccine uptake may be because of the concern around the speed of vaccine development and the use of novel mRNA platforms for COVID-19 vaccines. Conflicting communication strategies may also have contributed to parents experiencing decisional conflict or uncertainty about COVID-19 vaccination for children. Decisional conflict is the state of uncertainty about deciding and is common when the decision is based on choices that involve weighing risks and benefits [6]. Decisional conflict may be driven by questions about vaccine safety and effectiveness and disease severity, which underpin vaccine acceptance and uptake [7]. These issues were at the forefront for parents when considering COVID-19 vaccines for their children [8,9], as evidence and COVID epidemiology rapidly evolved throughout 2022. 

Decision support tools reduce decisional conflict and increase users’ informed participation in healthcare decisions requiring a trade-off between risks and benefits. Decision support tools include detailed decision aids, option grids, and value clarification exercises, which can be web-based, printed, or video-based. These tools can assist individuals by helping them express their decisions, educating them about their options and the risks and benefits associated with them and assisting in determining how these decisions align with personal values [8]. 

Decision support tools have been used to inform decision-making about a broad range of health conditions [10], and there is somewhat limited but promising evidence of their impact on vaccine decisions specifically. A 2021 systematic review of five randomized controlled trials found that decision aids can increase intention to vaccinate and reduce decisional conflict about vaccination [11]. A 2022 review of shared decision-making interventions, which included decision aids as well as interventions like conversation training for providers, found that such interventions increased vaccine uptake and decision confidence and could reduce decisional conflict [12]. 

At the start of the COVID-19 vaccine rollout for 5–11-year-old children in Australia, we conducted a comprehensive web search of Australian government and health websites. We found no decision support tools available for parents to guide COVID-19 vaccine decision-making, despite low levels of uptake for primary school children. Given the potential for decision support tools to benefit vaccine decision-making, we developed a simple, printable decision support tool that guides and supports parents about their decision to get their 5–11-year-old child vaccinated for COVID-19. The tool was designed to alleviate the decisional conflict experienced by some parents surrounding COVID-19 vaccines for children.

This study aimed to determine whether a short online decision support tool could improve parental decision-making about child COVID-19 vaccination. Our objectives were to assess whether the decision support tool could reduce parental decisional conflict and vaccine hesitancy and increase parental intention to vaccinate their 5–11-year-old child against COVID-19.

## 2. Materials and Methods

### 2.1. Study Design

This study applied a single-arm pre-post study design to evaluate the impact of a decision support tool using an online survey.

### 2.2. Participants and Setting

We used convenience and snowballing sampling methods [13], recruiting participants through paid Facebook advertisements and organic posts on Facebook and Instagram shared by the research team and their networks. Networks included the Murdoch Children’s Research Institute, the University of Melbourne, and Raising Children’s Network Facebook sites. Participants were encouraged to share the survey link with other prospective participants [13].

Participants were eligible if they met the following criteria: (1) currently living in the state of Victoria; (2) a parent or caregiver of a child aged 5–11 years; (3) their child had not yet received a COVID-19 vaccine, and (4) they did not intend to vaccinate their child.

A 15-min survey with 36 items (8 items before and 28 items after viewing the decision support tool) was hosted by REDCap [14,15]. Participants responded to the pre-intervention questions, viewed the tool, and immediately completed the post-intervention questions. Participants who answered only the pre-intervention questions were excluded as a change in study outcomes could not be determined.

Approval to conduct this research was provided by Royal Children’s Hospital Research Ethics and Governance following its ethics review and approval procedures [HREC 82021]. Consent was implied by survey completion.

All eligible participants who fully completed the survey between 9 May and 1 September 2022 were included in the study. 

### 2.3. Decision Support Tool

**Figure 1:**
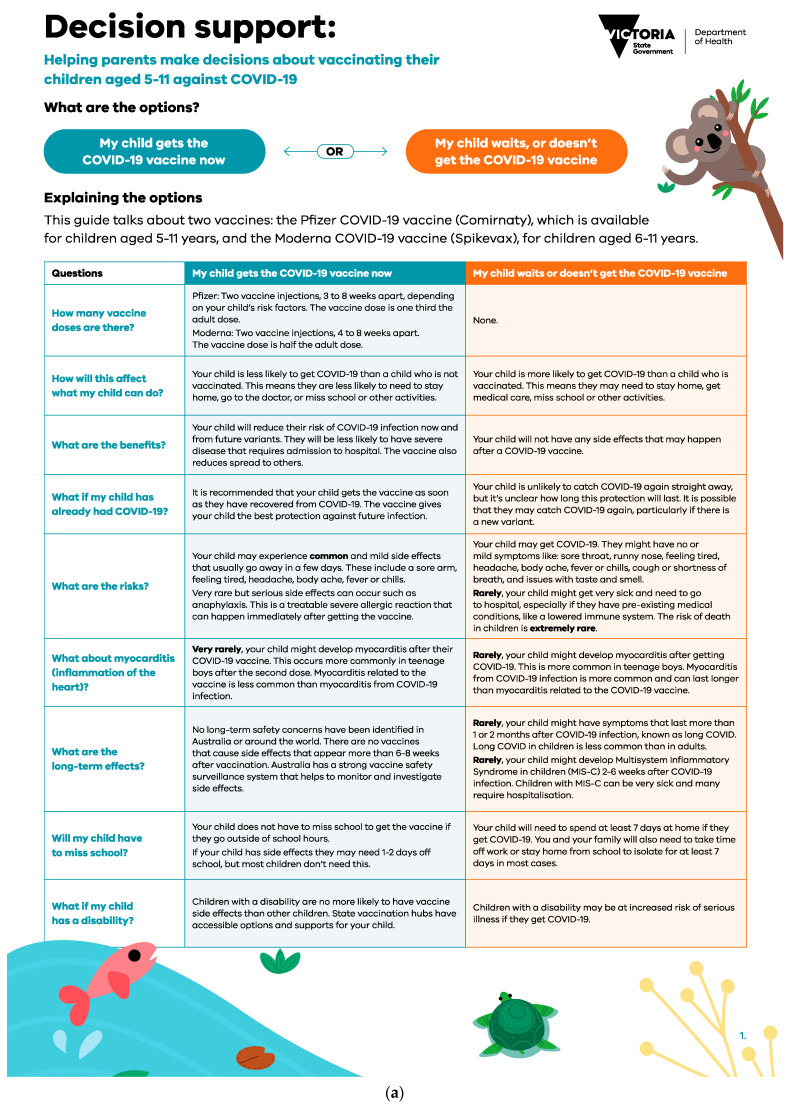

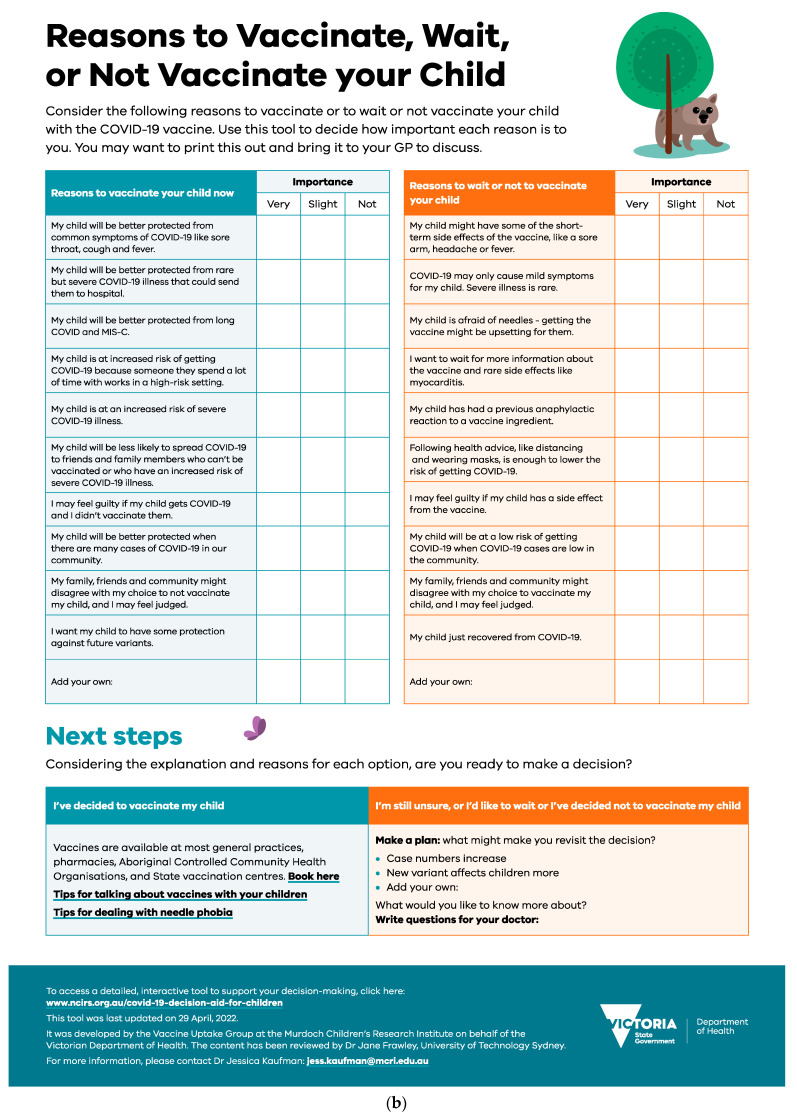
(**a**) Page 1 of the decision support tool. (**b**) Page 2 of the decision support tool.

The two-page decision support tool (Figure 1a,b) was designed by researchers with expertise in childhood infectious diseases, vaccination, and communication. It was reviewed for content validity by an external expert in decision support tools and vaccination. It was not feasible to pilot test the decision support tool due to the rapid development and implementation supporting the ongoing vaccine rollout. The tool presented balanced information about options to help them weigh the risks and benefits in the context of their values and addressed common questions and concerns held by parents at the time. The tool was designed to be used by parents with their healthcare professionals, where decision-making could be discussed as part of a consultation. Tools could be viewed online or printed. The first page of the tool contained an option grid outlining the outcomes associated with a parent’s choice to vaccinate versus waiting/not getting their child vaccinated. For example, the risks and benefits of getting vaccinated or waiting/not getting vaccinated. The second page gave parents a chance to clarify how important to them, on a scale of “not” to “very” important, different reasons to vaccinate/or not vaccinate their child. At the end of the tool, the possible next steps were outlined once the user had decided.

### 2.4. Outcomes and Measures

The primary outcome, decisional conflict, was measured using the validated 4-question “SURE test (Sure of myself; Understand information; Risk-benefit ratio; Encouragement)” for decisional conflict [16]. The four questions in the SURE test are relevant to the following stages of decision-making; (1) feeling certain about the decision, (2) feeling informed, (3) feeling clear about values, and (4) feeling supported in the decision [16] and have been used previously in studies evaluating the impact of vaccine decision aids on decisional conflict [17,18]. Each question is answered yes (1 point) or no (0 points), summed for a total score from 0–4. A score of less than 4 describes a person experiencing decisional conflict. 

We calculated the change in decisional conflict by subtracting individuals’ post-decisional conflict scores from their pre-decisional conflict scores. The difference in decision conflict score was categorised as increased, decreased, or remained the same if the participant’s decisional conflict was higher, lower, or unchanged after viewing the decision support tool compared to before for each SURE decisional conflict test question. Parents were categorised as having a positive change if they changed their answer from no to yes after viewing the tool; negative change if they changed from yes to no; and unchanged if their answer was the same before and after viewing the tool.

Intention to vaccinate their child was assessed by a question developed by the research team: “How likely is it that you will get a COVID-19 vaccine for your child aged between 5–11?” with response options on a 5-point Likert scale from definitely yes to not. Change in intention was categorised as increased, decreased or remained the same.

Hesitancy towards COVID-19 vaccines for children was assessed with the question: “How much do you agree with the following statement: ‘I feel hesitant about COVID-19 vaccines for children”, also developed by the research team. Response options were measured on a 4-point Likert scale from strongly agree to disagree and change in hesitancy strongly was categorised as increased, decreased or remained the same.

After viewing the decision support tool, parents rated their satisfaction with it. They provided demographic information including gender, age, education level, employment status, Indigenous status, culturally and linguistically diverse (CALD) status (defined as speaking a language other than English at home and/or if the participant is born outside of Australia), residential location (defined according to the Modified Monash Model as city, rural, remote or very remote) [19] and number of children. Time spent viewing the decision support tool was calculated through timestamps indicating how long each participant spent on the survey page containing the tool.

To gather additional insight into parents’ thoughts and feelings, parents could provide optional free-text responses to the following questions: “What is the main reason for your answer?” (Follow up to the question “How likely is it that you will get a COVID-19 vaccine for your child?” in the post-intervention section) and “Please provide any overall comments on how the resources could be improved”. 

### 2.5. Data Analysis

Categorical responses are presented as numbers and percentages. Wilcoxon matched-pairs signed-rank test was used to test the equality of matched pairs of observations. Binary logistic regression was used to investigate the association between reduction in decisional conflict and demographic characteristics. We used STATA statistical software version 17.0 for analysis. 

Free text responses were analysed using descriptive content analysis [20].

## 3. Results

### 3.1. Sample Description

**Figure 2:**
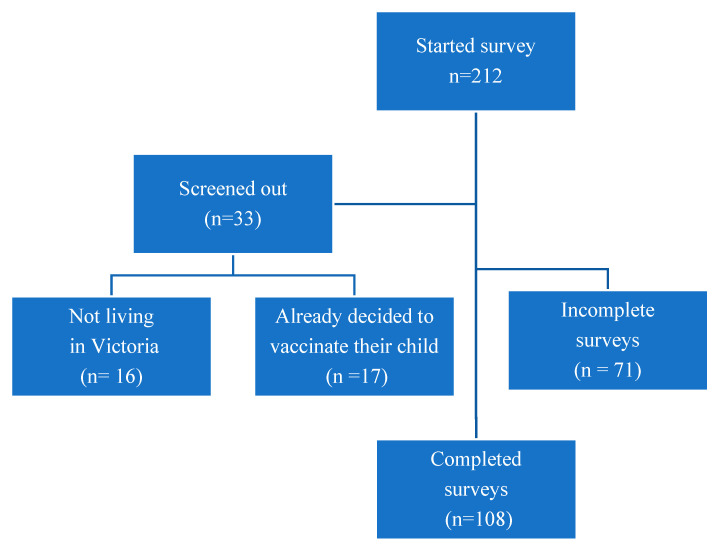
Study flow diagram.

A total of 212 individuals started the survey (Figure 2). Of these, 33 were screened out for not living in Victoria (16) or responding “definitely yes” regarding their intention to vaccinate their 5–11-year-old child (17). We excluded 71 participants who did not complete the post-intervention survey questions.

Demographic characteristics are summarised in Table 1. Most parents were female (92/108, 85%) and working full-time (58/108; 54%); 48% held a postgraduate degree (52/108), and 14% (15/108) were culturally and linguistically diverse and had a child with an at-risk medical condition (15/108). Almost one-third (34/108, 32%) of the parents had not received any doses of the COVID-19 vaccine themselves. 

### 3.2. Decisional Conflict

**Figure 3:**
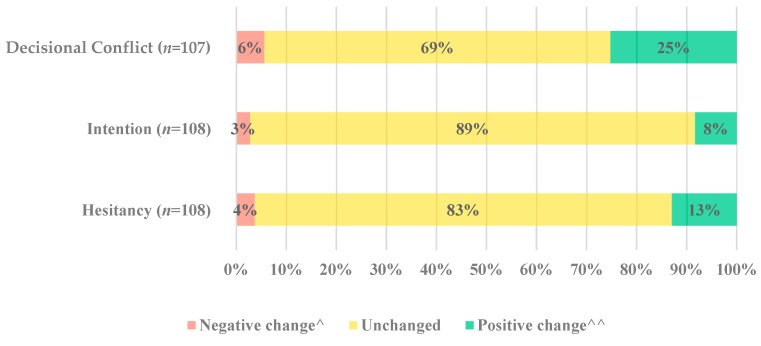
Change in decisional conflict, hesitancy, and intention: Percentage of parents with a positive, negative or no change in decisional conflict, intention, and hesitancy after viewing the decision support tool. ^ Negative change refers to an increase in decisional conflict and hesitancy and a decrease in intention. ^^ Positive change refers to a decrease in decisional conflict or hesitancy and an increase in intention Note: *n* = 107 for decisional conflict, as one participant did not answer one of the four questions.

There was statistical evidence of post-intervention difference in decisional conflict (Wilcoxon signed-rank test, *p*-value < 0.001). This was evident as one-quarter of parents (25%, 27/107) reduced their decisional conflict, whilst 6% (6/107) showed an increase in decisional conflict (Figure 3). Most parents had no change (69%, 74/107). 

**Figure 4:**
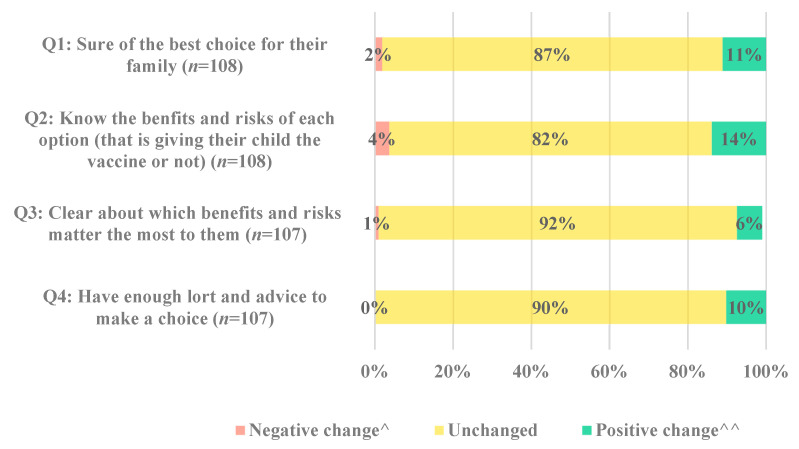
Decisional conflict by the SURE question: Proportion and number of participants who had a positive, negative or no change in their responses to each question in the SURE Test for Decisional Conflict after viewing the decision support tool. ^ Negative change refers to an increase in decisional conflict. Note: *n* = 107 for Q2, as one participant did not answer this question after viewing the tool. ^^ Positive change refers to a decrease in decisional conflict.

The change in response to each of the four decisional conflict questions is displayed in Figure 4. While most parents remained unchanged for each of the four questions, the proportion of parents with positive change was greater than those with negative change (Supplementary Figure S1). 

Decreased decisional conflict after viewing the decision support tool is associated with baseline intention to vaccinate, vaccine hesitancy and demographic characteristics of parents (Table 2). More parents (62%, 8/13) who were undecided about vaccinating their child reduced their decisional conflict, compared to parents (11.3%, 7/62) who did not intend to vaccinate their child (Odds Ratio (OR), 12.6 95% Confidence Interval (CI) 3.2 to 49.3). The tool reduced decisional conflict in 44% (20/45) of parents who had themselves received three doses of COVID-19 vaccine, compared to 3% (3/33) of parents who had not received any doses of COVID-19 vaccine (OR, 8.0 95% CI 2.1 to 30.1). For every additional 60 s spent viewing the decision support tool, the odds of experiencing reduced decisional conflict increased by 1.2 (95% CI 1.0 to 1.5). While there are uncertainties about the point estimates of the odds ratio due to the small sample size, the lower limits of the 95 CIs indicate sufficient evidence for each of these findings.

### 3.3. Intention and Vaccine Hesitancy

Before receiving the decision support tool, most parents reported that they would not get their child a COVID-19 vaccine (58% 63/108), while 12% (11/108) were unsure. Intention remained mostly unchanged (89% 96/108) pre- and post-intervention (Wilcoxon signed-rank test, *p*-value = 0.146, Figure 3). Before receiving the decision support tool, most parents reported hesitating about COVID-19 vaccines for children (92% 99/108). While the level of hesitancy towards COVID-19 vaccines for children remains unchanged for most parents (83% 90/108), there is statistical evidence of post-intervention change in hesitancy (Wilcoxon signed-rank test, *p*-value = 0.030, Figure 3). 

### 3.4. Reason for Intention

In the post-survey, 84% (78/108) of parents provided free text responses. Most responses were from people who were not or probably not likely to get their 5–11-year-old child vaccinated for COVID-19. We identified three main themes:

Safety: Parents felt insufficient long-term safety data for the COVID-19 vaccines, with one such parent saying, “*I would like to see some more long-term data*”. Other safety issues raised were children with allergies, such as a child who was anaphylactic to nuts, only getting the vaccine if it was mandatory.

Necessity: Some parents whose children had already had COVID-19 did not feel they needed to be vaccinated. Parents questioned the risk, benefit ratio of COVID-19 vaccines for children, often pointing to COVID-19 infection being mild in children. For example, one participant wrote, “*My unvaccinated child got COVID and was sick for two days. Children are not at risk.*” Another wrote, “*COVID is extremely mild for children*”.

Trust: Some parents would not vaccinate their children as they distrust the vaccine and data. One participant wrote, “*This poison will never enter my children’s bodies*”.

Written responses as to why parents were probably or going to get their child vaccinated included vaccines reducing the likelihood of catching COVID-19, clear reputable information and understanding the risks and benefits. For example, one participant wrote, “*I feel it will better protect my child and family from serious illness*”. 

### 3.5. Decision Support Tool Satisfaction

**Figure 5:**
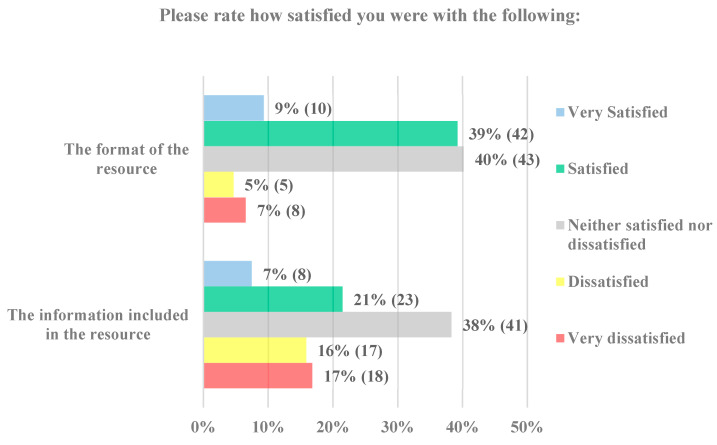
Resource format and information satisfaction levels of parents (*n* = 108).

Most parents responded that they were either satisfied (39% 42/108) or neither satisfied nor dissatisfied (40% 43/108) with the format of the decision support tool (Figure 5). When asked how satisfied they were with the information in the tool, one-third of parents were satisfied (21% 23/108) or neither satisfied nor dissatisfied (38% 41/108). Overall, parents were more satisfied with the format than the information.

## 4. Discussion

At the time of study in Australia in 2022, there were no decision support tools available for parents to guide their decision-making to vaccinate primary school children against COVID-19, despite low uptake levels. To address this gap, we developed and evaluated the impact of a COVID-19 vaccine decision support tool for parents of 5–11-year-old children. The decision tool reduced parents’ decisional conflict, with a quarter of parents having reduced decisional conflict post viewing the tool. This is consistent with other studies showing that decision support tools can effectively reduce decisional conflict in a vaccine context [10,21,22] and for other healthcare decisions [8,22]. The decision support tool had minimal impact on vaccine intention and hesitancy, with most parents remaining unchanged. The reasons parents provided for their low intention to vaccinate included negative concern about the risk-benefit equation of vaccination, wanting more long-term safety data, the child already having had COVID-19 infection and distrust in vaccines. Overall, people felt mostly satisfied or neutral about the format and information contained in the tool. 

The decision support tool was most effective at reducing decisional conflict in parents who were initially undecided about whether they would get their child vaccinated compared to those who were refusing. This highlights the importance of targeting interventions to individuals who are undecided or “sitting on the fence” about vaccination, as this group is more open to changing their behavior than individuals at either end of the vaccine hesitancy spectrum [23]. Parents who had been vaccinated for COVID-19 were also more likely to reduce their decisional conflict than those who had not been vaccinated. This aligns with other research, including a study by Horne et al., 2015 which found that an information intervention about measles vaccines was more likely to positively shift the attitudes of those initially more receptive to measles vaccination [24]. Compared to the COVID-19 vaccine coverage of Victorians aged 16 and over, our sample contained a high proportion of parents who themselves had not been vaccinated [25]. When the survey closed (1 September 2022), 96% of Victorians over 16 had received at least one dose of the COVID-19 vaccine. In contrast, only 69% of parents in our sample had at least one dose of the COVID-19 vaccine [25], suggesting that our sample was skewed towards vaccine refusers. This may have attenuated the support tool’s effect on decisional conflict, intention, and hesitancy. 

Parents’ reasons for low intention to vaccinate were comparable to the motivational barriers reported by parents internationally [26,27]. Parents with low intention predominantly cited concern about the safety of COVID-19 vaccines. This has been documented in vaccine-hesitant adults in Australia [28] and as a barrier for parents to have their child vaccinated with other childhood vaccinations [29]. 

Our findings should be viewed in the context of study design limitations. We could not pilot test the decision support tool or evaluation survey with parents as the tool was rapidly designed and implemented to address an urgent practical need and was provided to the Victorian Government. Participants were recruited using convenience sampling, which may have introduced self-section bias. The findings may not be generalisable beyond the study population. Participant recruitment and the survey were conducted online, which may have restricted participation from parents with lower internet literacy. Such parents may also need the most support as they may find it difficult to access vaccine resources online. The sample size for this study was relatively small despite efforts to recruit more parents and an extension in the data collection period to four months. This impacted the conclusions that could be drawn about the tool’s impact on intention and hesitancy in particular and the characteristics of the parents who may have the greatest potential benefit from the tool. However, similar sample sizes have been utilized by other studies with this primary outcome. Recruitment of parents attracted a high percentage of vaccine refusers who were unlikely to benefit from the decision support tool. We considered screening out parents who said they were not planning to vaccinate in addition to those who were. However, this would have limited the sample size further and prevented us from comparing the effectiveness of the decision support tool between undecided and refusing parents. The addition of a randomly sampled control group would have been a helpful comparator to determine the extent to which the sample was skewed by vaccine refusers. This should be considered for future studies investigating the uptake of contentious vaccines. A relatively large proportion (34%, 71/212) of parents who began the survey exited once they got to the decision support tool. Because demographic characteristics were captured at the end of the survey, we could not describe the characteristics of these individuals. It would be useful to know why these patients exited the survey and whether this was due to dissatisfaction or lack of comprehension of the decision support tool. Lastly, the quasi-experimental posttest design of this study makes it challenging to attribute the change in decisional conflict, intention, and hesitancy, albeit little, to the decision support tool or due to external factors. However, the immediate post-intervention assessment time point makes external influences unlikely. 

### Practice Implications

The dynamic nature of COVID-19 and changing vaccine recommendations led to confusion, and it was clear that many parents needed support with their decision-making. Recommendations for COVID-19 vaccines for children under 12 vary internationally. Some countries recommend vaccination [30,31], and others recommend vaccination only for children deemed as high risk [30,31]. In Australia, COVID-19 vaccination is recommended for all children over five years and high-risk children six months to five years. Despite this, uptake in the 5–11-year-old age group has been relatively low. Shared decision-making resources are a possible strategy to address concerns about COVID-19 vaccination [10] and improve uptake. 

For a decision support tool to be suitable for the needs of parents, we suggest the following recommendations. First, to address the constantly changing nature of the pandemic, emerging data about vaccine effectiveness and safety and circulating misinformation [7,32], the decision support tool should be updated with the latest data and address trending concerns. This could be achieved using an online editable platform linking to government recommendations or evidence summaries. This would also benefit parents who, like some in our study, are waiting for long-term vaccine safety data before deciding to vaccinate their child. Second, the tool should include links to scientific articles. Some parents in our study indicated that it would be useful for empirical data to be included to calculate the risks and benefits of vaccination better. These links should provide information for those who seek it but should not be included in full in the main text of the tool to avoid overloading people with detail. Third, decision support tools should be disseminated directly to healthcare providers, along with provider training in using the tool effectively to support shared decision-making. This training should include the suggestion that providers use the tool primarily with parents who have questions or are hesitant about the COVID-19 vaccine for their child but vaccinate themselves, as they are more open to choosing to vaccinate. 

## 5. Conclusions

Overall, this study adds to the evidence suggesting that decision support tools may be a relatively affordable and effective strategy to communicate the risks and benefits of vaccination, especially for those undecided about vaccination. Tailored decision support tools should be considered to reduce decisional conflict in other groups where individuals are undecided and experiencing decisional conflict about whether to vaccinate themselves or their child against COVID-19 or other vaccine-preventable diseases.

## Figures and Tables

**Table 1:** Characteristics of parents of children aged 5–11.

	Study Sample
Total Participants	108
	*n* (%)
Gender	
Male	8 (7.4)
Female	92 (85.2)
Neither or prefer not to say	8 (7.4)
CALD Status (born outside Australia and/or speaks a language other than English at home)	
No	93 (86.1)
Yes	15 (13.9)
The child (5–11 years old) has an underlying health condition	
No	93 (86.1)
Yes	15 (13.9)
Indigenous status	
No	98 (90.7)
Yes	1 (0.9)
Prefer not to say	9 (8.3)
Parent age	
18–29	1 (0.9)
30–39	52 (48.2)
40–49	52 (48.2)
50–59	3 (2.8)
Highest level of education	
High School	9 (8.3)
Trade certificate/diploma	13 (12.0)
Undergraduate degree	33 (30.6)
Postgraduate degree	52 (48.2)
None of the above	1 (0.9)
Employment status	
Part-time	36 (33.3)
Full-time	58 (53.7)
Retired	8 (7.4)
Not working and not seeking work (e.g., home caring duties)	1 (0.9)
Unemployed and seeking work	5 (4.6)
Residential location	
Metropolitan	94 (87.0)
Regional or rural	14 (13.0)
Number of children	
1	17 (15.7)
2	51 (47.2)
3	30 (27.8)
4+	10 (9.3)
Parent COVID-19 vaccine doses	
0	34 (31.5)
1	2 (1.9)
2	27 (25.0)
3	45 (41.7)
Time spent viewing decision support tool in seconds [median (IQ1–IQ3)]	67.6 (20.7–149.1)

**Table 2:** Decrease in decisional conflict by intention and demographics.

	Total Number of Participants	Experienced Decreased Decisional Conflict *n* (%)	Odds Ratio (95% CI)
Intention (at baseline)			
Not	62	7 (11.3%)	1
Probably not	26	9 (34.6%)	4.2 (1.4 to 12.9)
I’m not sure yet	13	8 (61.5%)	12.6 (3.2 to 49.3)
Probably yes	6	3 (50.0%)	7.9 (1.3 to 46.7)
Hesitancy (at baseline)			
Strongly agree	74	14 (18.9%)	1
Agree	24	11 (45.8%)	3.6 (1.4 to 9.8)
Disagree	5	2 (40.0%)	2.9 (0.4 to 18.8)
Strongly disagree	4	0 (0%)	n/a *
Gender			
Male	8	1 (12.5%)	1
Female	92	24 (26.1%)	2.5 (0.3 to 21.1)
Neither or prefer not to say	7	2 (28.6%)	2.8 (0.2 to 40.1)
CALD Status (born outside Australia and/or speaks a language other than English at home)			
Yes	14	3 (21.4%)	1
No	93	24 (25.8%)	1.3 (0.3 to 5.0)
Parent COVID-19 vaccination status			
Not vaccinated	33	3 (3%)	1
1	2	0 (0%)	n/a *
2	27	4 (14.8%)	1.7 (0.4 to 8.6)
3	45	20 (44.4%)	8.0 (2.1 to 30.1)
Highest level of education			
High School	9	2 (22.2%)	1
Trade certificate/diploma	13	2 (15.4%)	0.6 (0.1 to 5.6)
Undergraduate degree	32	9 (28.1%)	1.4 (0.2 to 7.9)
Postgraduate degree	52	14 (26.9%)	1.3 (0.2 to 7.0)
None of the above	1	0 (0%)	n/a *
Employment status			
Part-time	35	6 (17.1%)	1
Full-time	58	17 (29.3%)	2.0 (0.7 to 5.7)
Retired	8	3 (37.5%)	2.9 (0.5 to 15.6)
Unemployed	6	1 (16.7%)	1.0 (0.1 to 9.8)
Number of children			
1	17	2 (11.8%)	1
2	51	15 (29.4%)	3.1 (0.6 to 15.4)
3	29	7 (24.1%)	2.4 (0.4 to 13.1)
4+	10	3 (30.0%)	3.2 (0.4 to 23.8)
Parent’s age			
<40	53	14 (26.4%)	1
≥4	54	13 (24.1%)	0.9 (0.4 to 2.1)
The child (5–11 years old) has an underlying health condition			
Yes	15	5 (33.3%)	1
No	92	22 (23.9)	0.6 (0.2 to 2.0)
Duration of time spent viewing the decision support tool (per 60 s increase in period)	107	Mean (SD) 106.2 (121.7)	1.2 (1.0 to 1.5)

Note *n* = 107 as one participant did not answer one of the decisional conflict questions in the Post section. * Note no individuals within this group had a decrease in decisional conflict; therefore, no odds ratio can be calculated.

## Data Availability

Restrictions apply to the availability of these data. Data are available from the authors upon request.

## Supplementary Materials

The following supporting information can be downloaded at: https://www.mdpi.com/article/10.3390/vaccines11081296/s1, Figure S1: Decisional conflict by SURE question—Proportion and number of “Yes” responses.
