# Supplementary figures and images for: Reducing COVID-19 Vaccine Decisional Conflict in Parents of 5–11-Year-Old Children in Australia: A Single Arm Pre-Post Study
Source: Vaccines (Basel). 2023 Jul 28;11(8):1296. doi: 10.3390/vaccines11081296 (PMC10458235; doi:10.3390/vaccines11081296)

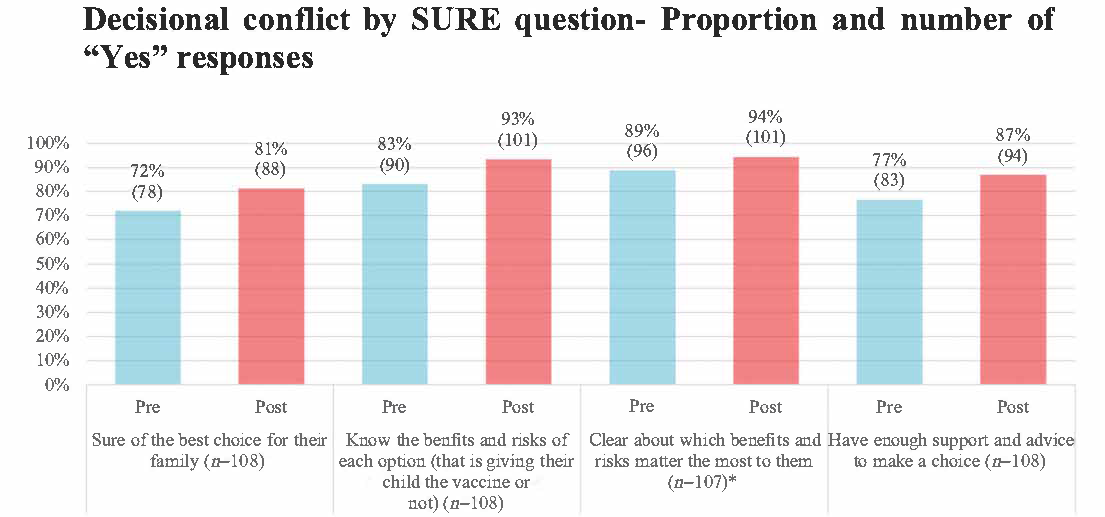

Supplement: Supplementary file 1 [file vaccines-11-01296-s001.zip › vaccines-2452984-supplementary.png]
